# An innovative MGM–BPNN–ARIMA model for China’s energy consumption structure forecasting from the perspective of compositional data

**DOI:** 10.1038/s41598-024-58966-z

**Published:** 2024-04-11

**Authors:** Ruixia Suo, Qi Wang, Yuanyuan Tan, Qiutong Han

**Affiliations:** College of Management, Xi’an University of Science and Technology, Xi’an, 710054 China

**Keywords:** Aitchison distance, Compositional data, Combined model, Energy consumption structure, Environmental social sciences, Energy and society, Energy policy

## Abstract

Effective forecasting of energy consumption structure is vital for China to reach its “dual carbon” objective. However, little attention has been paid to existing studies on the holistic nature and internal properties of energy consumption structure. Therefore, this paper incorporates the theory of compositional data into the study of energy consumption structure, which not only takes into account the specificity of the internal features of the structure, but also digs deeper into the relative information. Meanwhile, based on the minimization theory of squares of the Aitchison distance in the compositional data, a combined model based on the three single models, namely the metabolism grey model (MGM), back-propagation neural network (BPNN) model, and autoregressive integrated moving average (ARIMA) model, is structured in this paper. The forecast results of the energy consumption structure in 2023–2040 indicate that the future energy consumption structure of China will evolve towards a more diversified pattern, but the proportion of natural gas and non-fossil energy has yet to meet the policy goals set by the government. This paper not only suggests that compositional data from joint prediction models have a high applicability value in the energy sector, but also has some theoretical significance for adapting and improving the energy consumption structure in China.

## Introduction

### Background and motivation

As the global greenhouse effect intensifies, how to effectively address climate change has become a global issue for all nations^1,2^. The Paris Agreement, a legally binding climate protocol outlining long-term development goals for future temperatures, was signed by about 200 nations in 2015^3^. Hence, a growing number of nations have developed pertinent national strategies with an ambition for a carbon-free future^4^. As the largest energy consumer and carbon emitter in the world, the Chinese government has committed to reaching carbon neutrality by 2060, and implementing “stronger and more powerful policies and measures” to achieve the peak of emissions by 2030^5^. It implies that China will face great challenges in reducing emissions in the future, and that formulating effective “dual carbon” development strategies is one of the priorities of the Chinese government.

Energy consumption is the majority source of carbon emissions in China^6^. Furthermore, China has developed an energy consumption pattern that is dominated by fossil fuels due to its resource structure, which is characterized as “rich in coal, poor in oil, and short of gas”^7^. Nevertheless, the combustion of fossil energy (e.g., coal and oil) generates significant amounts of carbon dioxide emissions. As a result, it is crucial to adjust and optimize the energy consumption structure for China to reduce carbon emissions.

**Figure 1:**
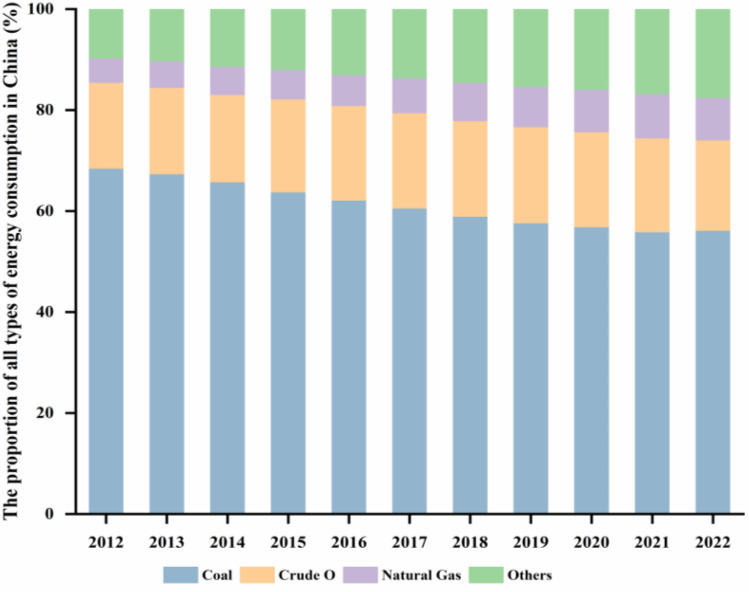
The structure of energy consumption in China during 2012–2022. *Note: Data from China National Statistical Yearbook.

The energy consumption structure is mostly made up of four categories: coal, crude oil, gas, and others (e.g., hydroelectric power, nuclear power)^8^. As shown in Fig. 1, fossil energy has historically dominated the energy consumption structure in China, and yet its percentage is decreasing every year, while other clean energy (e.g., natural gas and hydropower) is increasing. Actually, the Chinese government’s series of emission reduction initiatives have been more effective, and the energy consumption structure is optimally adjusted each year. However, it is unknown whether the current emissions reduction initiatives will achieve the government’s stated policy goals as expected. Therefore, effectively forecasting the trend of energy consumption structure can not only verify the feasibility of existing policies, but also facilitate the adjustment and formulation of related policies, which can boost the government’s ability to govern.

### Literature review

Due to the inherent complexity and asymmetry of multiple interacting elements, energy consumption forecasting has become a challenging problem in the field of time forecasting^9^. At present, there are numerous studies in the energy field related to the dynamical evolution of the energy structure. The quantitative research methods used by scholars fall into two main categories: univariate forecasting models and multivariate forecasting models.

Univariate forecasting models in energy consumption are mainly based on raw series data for forecasting studies, without the intervention of additional influencing factors^10^. In particular, autoregressive integrated moving average (ARIMA) model and grey model (GM) are most widely used in energy consumption forecasting^11,12^. Jiang et al.^13^ estimated coal costs, consumption, and investment for 2016–2030 in China. By using an ARIMA model, Akram et al.^14^ applied an ARIMA model to forecast the residential energy consumption in the household sector, which belongs to the Eurozone countries. Ding et al.^15^ proposed a structural adaptive grey model with adjustable temporal power terms to address the time series nonlinear problem of nuclear energy consumption. Yuan et al.^16^ projected the primary energy consumption in China using the ARIMA, GM(1,1), and GM-ARIMA hybrid models. Meanwhile, Li et al.^17^ developed two combined models: the metabolism grey model with autoregressive integrated moving average model (MGM-ARIMA), and the back-propagation neural network with autoregressive integrated moving average model (BPNN-ARIMA) for forecasting energy consumption in India during 2018–2030. Ma and Wang^18^ constructed a nonlinear grey model-autoregressive integrated moving average model (NGM-ARIMA) to forecast the energy consumption in South Africa during 2017–2030.

Multivariate forecasting models in energy consumption, which mainly refer to the construction of forecast models by exploring the relevant influencing factors^16^. There are numerous external influencing factors affecting energy consumption forecasting, and how to identify the furthest relevant factors from the vast potential factors is the crucial issue in the perspective of this study^19^. Scholars have adopted various methods to explore the influencing factors, such as logarithmic mean divisia index (LMDI) method^20^ and stepwise regression^21^. Simultaneously, within this study perspective, artificial intelligence algorithms^22^, like support vector machines^23^ and neural networks^24^, are frequently employed for energy forecast. Xia and Wang^25^ solved the contribution values of the influencing forces affecting the energy consumption structure by the LMDI method, and used an empirical model decomposition model to break down the influencing factors with large contribution values into modal components at various scales. According to the LMDI method, Chai et al.^26^ classified influencing drivers of gas consumption into the indicators of economic progress and cleanliness, and constructed a stochastic impacts by regression on population, affluence, and technology (STIRPAT) model, combined with partial least squares regression (PLSR) to analyze the scenario of natural gas consumption in China during 2016–2025. He et al^19^ utilized the stepwise regression method to identify major influencing factors and developed two probability density forecasting methods to estimate the consumption of energy in Anhui Province during 2015–2023, Liu et al^27^ used LMDI method to analyze the driving factors of carbon emission in Beijing, Tianjin, Shanghai and Chongqing.

Based on the aforementioned diverse literature, it is clear that the majority of current research has focused on the absolute amount of specific types of energy consumption, while too few studies have examined the relative information underlying the totality of energy consumption, which also implies that there are seldom studies that consider energy consumption structure for a whole^28^. The energy consumption structure is essentially a holistic system and should jointly take into account the variability among energy types^29^. Because the energy consumption structure is comprised of four energy sub-structures: coal, oil, natural gas, and other energy resources, which are required to satisfy non-negativity and the total sum should be one^30^. However, the study of energy consumption structure using traditional models does not fully investigate the relative information behind the entire structure and overlooks the holistic nature of the structure. To address this research issue, this paper incorporates the theory of compositional data into the investigation of the energy consumption structure.

Compositional data is a class of complex data with a special structure, which mainly describes the relative information among the components rather than their absolute values, and for which every knowledge about the components must be based on the ratio^31^. The basic concept behind modeling on compositional data is that the initial data is first transformed to produce intertemporal bisectional variables using appropriate techniques. And intermediate variables are then modeled and manipulated using basic modeling methods. Finally, the data results are reduced to compositional data by corresponding inverted transformations^32,33^. Recently, the compositional data theory has been applied extensively to forecast the regional industrial and economic structure^34^, study the shift of population structure^35^, and analyze the distribution of rock composition^36^, which are already successfully implemented in numerous areas including agriculture, economics and geology, but it is used less in the energy sector. Qian et al.^30^ suggested a unique adaptive discrete grey forecasting model based on compositional data. He et al.^28^ developed a dimension reduction through hyperspherical transformation and composite quantile regression neural network (DRHT-CQRNN) model to forecast the structure of total energy consumption in Chongqing during “the 14th Five-Year Plan period (2021–2025)”. Zhang et al.^37^ forecasted the structure of bioenergy generation in China based on an innovative grey compositional data model.

At present, few scholars consider the energy consumption structure as a whole system, and the relative information on its constituent components is lacking. Therefore, the theory of compositional data is introduced into the energy consumption structure in this paper to thoroughly explore the internal features of the structure and its interrelations. At the same time, it is found that all the current studies of energy consumption structure based on compositional data are single-mode models. In contrast, the combined model can combine the advantages of each single forecast model to enhance its overall accuracy of forecast and make the model fit and forecast extremely stable^38,39^. Therefore, a joint model is constructed based on the theory of compositional data in this paper. The key to the development of a comprehensive model is to determine the single model weights, yet weight selection is a major challenge for combined forecasting methods^40^. There are additional common methods for determining the weights of the combined model, such as minimization of the sum of squares error^41^ and reciprocal variance method^42^. However, since the compositional data is merely a vector, calculating its inaccuracy cannot be done by directly deducting the true value from the predicted value; On the contrary, all its internal features must be sufficiently considered. Therefore, the distance between vectors of compositional data is used as a measure of prediction error in this paper, whereby the weighting factor is derived from the minimum squared sum of the Atchison distance among the forecast and true values, then the combined MGM-BPNN-ARIMA model base on compositional data is proposed to forecast the energy consumption structure of China in 2023–2040.

### Contribution and research structure

The following are the contributions of this paper.

1. At present, few studies have considered an energy consumption structure as a whole system, while the relative information about the components of the structure has not been adequately explored. In this paper, we introduce the theory of compositional data into the energy consumption structure and systematically consider the internal features of the energy consumption structure, which fully satisfies the requirement of the non-negative and constant sum of its components.
2. Considering the vector property of the compositional data, therefore, based on the Aitchison distance sum of squares theory, this paper proposes a combined MGM-BPNN-ARIMA model based on the DRHT method, which has higher prediction accuracy than a single model.
3. Compare the model forecast results with the current policy goals proposed by the Chinese government. It is conceivable to predict whether China will meet its policy objectives on time, and to make relevant practical recommendations to legislators.

The remainder of the paper is organized as follows. "Materials and methods" section describes the methodology involved in this paper, which includes the theory of compositional data and methods for combining forecast models on compositional data; "Model establishment and analysis" section explains the construction of the specific MGM-BP-ARIMA merger model; "Forecast results and discussion" section contains the results and analysis of the forecast for China’s energy consumption structure during 2023–2040; "Conclusions" section presents the relevant conclusions.

## Materials and Methods

### Methodology

**Figure 2:**
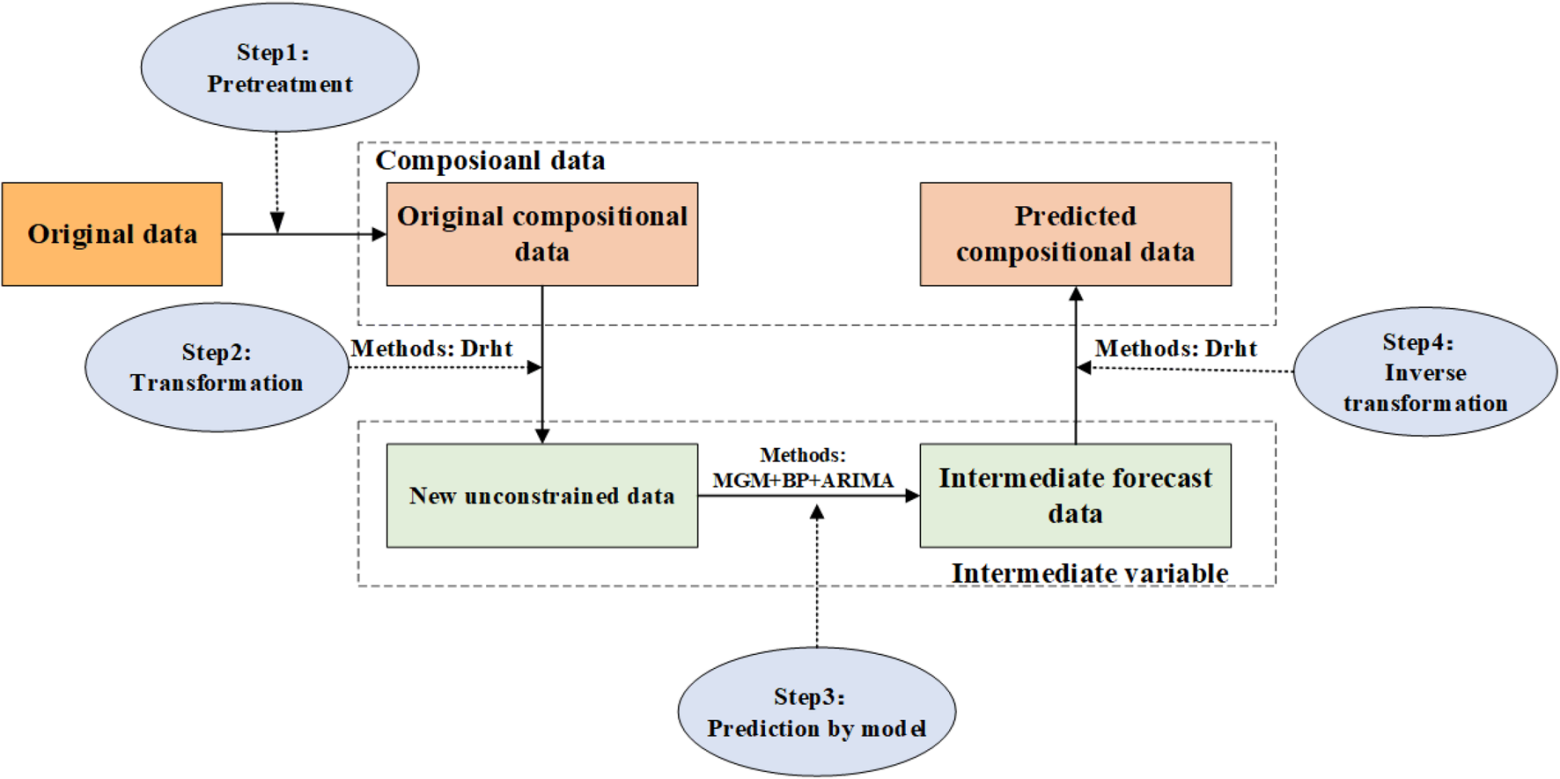
The basic process of compositional data forecast.

The compositional data are positive data that solely provide relative information, which adds up to a constant in the majority of instances^43^. Figure 2 illustrates the major process of the forecast study from the perspective of compositional data involved in this paper, which is classified into the following four primary steps.

*Step 1*: Take the correlation operation to turn the original data into compositional data (the data are mutually constrained and the sum ratio is distinct).

*Step 2*: Use appropriate transformation techniques to create original unconstrained variables from compositional data.

*Step 3*: Adopt a correlated time series prediction model for the original variables.

*Step 4*: The inverse transformation technique corresponding to the conversion method in Step 2 is taken to convert to the final desired compositional data values.

#### Compositional data

To represent a series of compositional data, each value of *x*_*i*_ (*i* = 1, 2, …, D) is consistently greater than 0 and satisfies $\sum\limits_{{{\text{i = }}1}}^{D} {x_{i} { = }c}$, where c is a constant. Therefore, the space *S*^*D*^ formed by compositional data satisfying all the above conditions can be described as follows.

1 $$S^{D} = \left\{ {X = \left[ {x_{1} ,x_{2} ,...,x_{D} } \right]:x_{i} > 0,i = 1,2,...,D;\sum\limits_{i = 1}^{D} {x_{i} = c} } \right\}$$

The elements are in *D*-dimensional row vectors, but since the sum of the components is fixed, that makes it a vector space of *D*-1 dimensions.

Nevertheless, due to the fixed-sum constraint of the compositional data, typical statistical approaches cannot be directly applied to the mathematical evaluation of compositional data^44^. To overcome the limitations associated with compositional data transformations for general statistical analysis, Aitchison^31^ proposed a logistic normal distribution model, and addressed the fixed-sum constraint problem with log-ratio transformation method. Egozcue et al.^32^ put forward the isometric log-ratio transformation to handle overlapping subcomponents in compositional decomposition. However, all of the above approaches require the components to be nonzero, which presents certain drawbacks. To further solve the zero-component problem, Wang et al.^45^ proposed a dimensionality reduction by the hyper spherical transformation, effectively resolving the dilemma of the existence of zero components in the compositional transformation. The application of spherical coordinate transformation to practical time series forecasting is described as follows.

Set $X = \left[ {x_{1} ,x_{2} ,...,x_{D} } \right]$ is a composition vector, which satisfies:

2 $$\sum\limits_{i = 1}^{D} {x_{i} = 1} ,0 \leqslant x_{i} \leqslant 1$$

If each component of the compositional vector is treated with a square root, then the following results can be obtained:

3 $$y_{i} = \sqrt {x_{i} } \left( {i = 1,2,...,D} \right)$$

And it also means that: $\sum\limits_{i = 1}^{D} {y_{i}^{2} } = 1$.

the vector $Y = \left[ {y_{1} ,y_{2} ,...,y_{D} } \right]$ can be regarded as a point on the hypersphere. The spherical coordinate transformation maps the D-dimensional vector $Y = \left[ {y_{1} ,y_{2} ,...,y_{D} } \right]$ to the hypersphere $\left[ {r,\theta_{1} ,\theta_{2,} ...,\theta_{D} } \right]$, where can be satisfied with $r^{2} = ||y||^{2} = 1$.

Thus, the computation procedure of the Drht can be summarized as follows:

4 $$\left\{ {\begin{array}{*{20}l} {\theta_{D} = {\text{arc}}\cos y_{D} } \hfill \\ {\theta_{D - 1} = {\text{arc}}\cos \left( {\frac{{y_{D - 1} }}{{\sin \theta_{D} }}} \right)} \hfill \\ {\theta_{D - 2} = {\text{arc}}\cos \left( {\frac{{y_{D - 2} }}{{\sin \theta_{D} \sin \theta_{D - 1} }}} \right)} \hfill \\ {...} \hfill \\ {\theta_{2} = {\text{arc}}\cos \left( {\frac{{y_{2} }}{{\sin \theta_{D} \sin \theta_{D - 1} \cdots \sin \theta_{3} }}} \right)} \hfill \\ \end{array} } \right.$$

The calculation process of Drht inverse transformation can be summarized as follows:

5 $$\left\{ {\begin{array}{*{20}l} {y_{1} = \sin \theta_{2} \sin \theta_{3} \sin \theta_{4} \cdots \sin \theta_{D} } \hfill \\ {y_{2} = \cos \theta_{2} \sin \theta_{3} \sin \theta_{4} \cdots \sin \theta_{D} } \hfill \\ {y_{3} = \cos \theta_{3} \sin \theta_{4} \cdots \sin \theta_{D} } \hfill \\ {...} \hfill \\ {y_{D - 2} = \cos \theta_{D - 2} \sin \theta_{D - 1} \sin \theta_{D} } \hfill \\ {y_{D - 1} = \cos \theta_{D - 1} \sin \theta_{D} } \hfill \\ {y_{D} = \cos \theta_{D} } \hfill \\ \end{array} } \right.$$

#### Single model

1. MGM model

Grey model theory^46^ was proposed by Professor Deng Julong to solve the information uncertainty within the system. GM(1,1) model is an essential component of grey system theory, which is concerned with forecasting small sample data by incomplete information. The essential concept of the GM(1,1) model is to generate the primary series by one accumulation, and then create a differential equation model to roughly obtain an approximate estimate of the original series, so as to forecast the subsequent development of the original data. The specific process of modeling the GM (1,1) model is as follows.

*Step 1*: Conduct an addition of the initial sequence $X^{\left( 0 \right)} = \left\{ {X^{\left( 0 \right)} \left( 2 \right),X^{\left( 0 \right)} \left( 3 \right),...X^{\left( 0 \right)} \left( n \right)} \right\}$ to obtain the new sequence $x^{\left( 1 \right)}$ (the One-AGO sequence $x^{\left( 0 \right)}$).

6 $$x^{\left( 1 \right)} \left( m \right) = \sum\limits_{i = 1}^{m} {x^{\left( 0 \right)} \left( i \right),i = 1,2,...,} n$$

*Step 2*: Compute the mean of the immediate neighbors of the series $x^{\left( 1 \right)}$ to generate the series $z^{\left( 1 \right)} = \left( {z^{\left( 1 \right)} \left( 2 \right),z^{\left( 1 \right)} \left( 3 \right),...,z^{\left( 1 \right)} \left( n \right)} \right)$.

7 $$z^{\left( 1 \right)} \left( m \right) = \frac{1}{2}x^{\left( 1 \right)} \left( m \right) + \frac{1}{2}x^{\left( 1 \right)} \left( {m - 1} \right),m = 2,3,..n$$

*Step 3*: Construct the whitening differential equation for GM(1,1) based on the above formula.

8 $$\frac{{dx^{\left( 1 \right)} \left( t \right)}}{dt} + ax^{\left( 1 \right)} \left( t \right) = b$$

where *b* denotes the amount of ash action and *-a* denotes the development factor.

*Step 4*: Introduce matrix form to calculate the data matrices *B* and* Y*.

9 $$B = \left[ {\begin{array}{*{20}c} { - z^{\left( 1 \right)} \left( 2 \right)} \\ { - z^{\left( 1 \right)} \left( 3 \right)} \\ {...} \\ { - z^{\left( 1 \right)} \left( n \right)} \\ \end{array} } \right],\;Y = \left[ {\begin{array}{*{20}c} {x^{\left( 0 \right)} \left( 2 \right)} \\ {x^{\left( 0 \right)} \left( 3 \right)} \\ {...} \\ {x^{\left( 0 \right)} \left( n \right)} \\ \end{array} } \right]$$

*Step 5*: Apply the least square method on the estimates of the parameters a and *b*.

10 $$\hat{u} = \left( {\begin{array}{*{20}c} {\hat{a}} \\ {\hat{b}} \\ \end{array} } \right) = \left( {B^{T} B} \right)^{ - 1} B^{T} Y$$

*Step 6*: Substitute the solved $\hat{a},\hat{b}$ into the whitening differential equation, to derive the time-responsive function of the differential equation.

11 $$\hat{x}^{\left( 1 \right)} \left( {m + 1} \right) = \left[ {x^{\left( 0 \right)} \left( 1 \right) - \frac{{\hat{b}}}{{\hat{a}}}} \right]e^{{ - \hat{a}m}} + \frac{{\hat{b}}}{{\hat{a}}},m = 1,2,...,n - 1$$

*Step 7*: Perform the cumulative subtraction operation to obtain the original sequence $x^{\left( 0 \right)}$ of the predicted value.

12 $$\hat{x}^{\left( 0 \right)} \left( {m + 1} \right) = \hat{x}^{\left( 1 \right)} \left( {m + 1} \right) - \hat{x}^{\left( 0 \right)} \left( m \right),m = 0,1,...,n$$

By continuously adding new information, while removing old information promptly, the modeling sequence will more closely represent the present features of the system. In practical forecasting, as the system grows, the information significance of the old data will gradually decline. The MGM(1,1) model is a modernized version of the conventional grey model^17^. Its forecast principle is to utilize the latest data $X^{\left( 0 \right)} \left( {k + 1} \right)$ predicted by the GM(1,1) model, to replace the oldest data $X^{\left( 0 \right)} \left( 1 \right)$ in the primary data series $X^{\left( 0 \right)}$, to maintain the dimensionality of the data series. Then the GM(1,1) model is repeated with the newest data series $X^{\left( 0 \right)} = \left\{ {X^{\left( 0 \right)} \left( 2 \right),X^{\left( 0 \right)} \left( 3 \right),...X^{\left( 0 \right)} \left( {k + 1} \right)} \right\}$, and the new data $X^{\left( 0 \right)} \left( {k + 2} \right)$ is added to $X^{\left( 0 \right)} = \left\{ {X^{\left( 0 \right)} \left( 2 \right),X^{\left( 0 \right)} \left( 3 \right),...X^{\left( 0 \right)} \left( {k + 1} \right)} \right\}$ and then subtracted from $X^{\left( 0 \right)} \left( 2 \right)$ (forming a new series), and the GM (1,1) model will then be used again for forecast and testing. Continue in this manner until the prediction target.

(2) BPNN model

The BPNN model is a multilayer feedforward neural contraction network model trained by a back-ward error propagation algorithm^47^. The propagation of the signal and the subsequent propagation of the mistake make up the bulk of the training process. Firstly, the activation function is weighted to calculate the signal in the input layer, which is then sent to the hidden layer and will ultimately be propagated to the output layer as well. If the requirements of the model error are not met, the weights and thresholds of the BPNN are continuously adjusted based on the gradient descent method, which entails the normal input of the signal again, and the cycle repeats until the output signal obtained from the output layer, which fulfills the accuracy requirements of the model.

(3) ARIMA model

The ARIMA model was originally introduced by Box and Jenkins in the early 1970s as a time series forecasting method. It has found applications in statistics and computational economics, where it is known to be the most widely employed model for time series forecasting. The AR, MA, and ARMA models are the main models used with this model. Essentially, the ARIMA model employs differencing to first smooth the non-stationary data before applying the ARMA model to the stationary data. Moreo-ver, the ARMA model is made up of two components: the AR model and the MA model^48^.

The equation of the AR(p) model is defined as:

13 $$y_{t} = \mu + \sum\limits_{i = 1}^{p} {\gamma_{i} y_{t - i} } + \varepsilon_{t}$$

The equation for the MA(q) model is defined as:

14 $$y_{t} = \mu + \sum\limits_{i = 1}^{q} {\theta_{i} \varepsilon_{t - i} } + \varepsilon_{t}$$

The equation for the ARMA(p, q) model is defined as:

15 $$y_{t} = \mu + \sum\limits_{i = 1}^{p} {\gamma_{i} y_{t - i} } + \varepsilon_{t} + \sum\limits_{i = 1}^{q} {\theta_{i} \varepsilon_{t - i} }$$

where $\mu$ is the constant term, $\gamma_{i}$ is the AR model coefficient, $\theta_{i}$ is the MA model coefficient, $\varepsilon_{t}$ is the white noise series, $p$ is the autoregressive of orders, and $q$ is the moving average of orders.

#### Combined Model

By setting appropriate weights and integrating the projections in a weighted manner, a combined model incorporates forecasts obtained from individual forecasting method. Leveraging the model construction based on each single model, the combined model can maximize the information utilization and thus can optimize the forecast results substantially. The mathematical formulation for the combined model is represented by the following expression.

16 $$\left\{ {\begin{array}{*{20}l} {f\left( t \right) = \sum\limits_{i = 1}^{n} {\omega_{i} \hat{f}_{i} \left( t \right)} } \hfill \\ {s.t{\mkern 1mu} {\mkern 1mu} \sum\limits_{i = 1}^{n} {\omega_{i} } = 1} \hfill \\ \end{array} } \right.$$

where $\hat{f}_{i} \left( t \right)$ is the prediction value for the *i*th method at moment *t* and $\omega_{i} \left( t \right)$ is the combined weight for the *i*th model at the moment *t*.

Since the compositional data is just a vector, calculating its inaccuracy cannot be done by simply deducting the true value from the forecast value; instead, all of its internal characteristics must be fully considered. Therefore, in this paper, the distances among the vectors of the compositional data are utilized as a measure of forecast error, whereby the weighting factor is derived from the minimum squared sum of the Atchison distance among the forecast and true values. The Atchison distance is a critical dimension of the compositional data, since it reflects the difference between the proportions of the data. The Acheson distance is defined as follows.

For any $x,y \in S^{D}$, then the Aitchison distance between *x* and *y* would be equal to:

17 $$d_{S} (x,y) = \sqrt {\sum\limits_{{i{ = }1}}^{D} {\left( {\ln \frac{{x_{i} }}{g\left( x \right)} - \ln \frac{{y_{i} }}{g\left( y \right)}} \right)^{2} } }$$

18 $$g\left( x \right) = \sqrt[D]{{\prod\limits_{{i{ = }1}}^{D} {x_{i} } }},g\left( y \right) = \sqrt[D]{{\prod\limits_{{i{ = }1}}^{D} {y_{i} } }}$$

The error value of the combined prediction of the compositional data for weight calculation based on the Aitchison distance sum of squares is expressed as:

19 $$J = \sum\limits_{t = 1}^{T} {d_{S}^{2} \left( {x^{t} ,\hat{x}^{t} } \right)} = \sum\limits_{t = 1}^{T} {\sum\limits_{i = 1}^{D} {\left( {\ln \,\frac{{x_{i}^{t} }}{{g\left( {x^{t} } \right)}} - \ln \,\frac{{\hat{x}_{i}^{t} }}{{g\left( {\hat{x}_{i}^{t} } \right)}}} \right)}^{2} }$$

The error value of a single prediction model for each compositional data point at t is:

20 $$e_{it} = \left[ {\ln \,\frac{{x_{i}^{t} }}{{g\left( {x^{t} } \right)}} - \ln \,\frac{{\hat{x}_{1i}^{t} }}{{g\left( {\hat{x}_{1}^{t} } \right)}},\ln \,\frac{{x_{i}^{t} }}{{g\left( {x^{t} } \right)}} - \ln \,\frac{{\hat{x}_{2i}^{t} }}{{g\left( {\hat{x}_{2}^{t} } \right)}},...,\ln \,\frac{{x_{i}^{t} }}{{g\left( {x^{t} } \right)}} - \ln \,\frac{{\hat{x}_{ni}^{t} }}{{g\left( {\hat{x}_{n}^{t} } \right)}}} \right]^{T}$$

The error sum of squares for each single compositional data prediction model is expressed as:

21 $$E = \sum\limits_{t = 1}^{T} {\sum\limits_{i = 1}^{D} {e_{it} e_{it}^{T} } }$$

According to the above equations, the combined model error can be expressed as:

22 $$J = \alpha^{T} E\alpha$$

where $\alpha$ denotes the weighted coefficient vector of the combined model.

By introducing the *n*-dimensional vector $R = \left[ {1,1,..,1} \right]^{T}$, the constraint on the weighting coefficients can be expressed as:

23 $$R^{T} \alpha = 1$$

With the aforementioned transformation, the key to solving for the weights based on the Aitchison distance sum of squares is to achieve *J* minimization while introducing the Lagrange multiplier. To minimize *J*, the first-order partial derivative of *J* concerning being zero, and the final weight coefficient can be calculated as follows.

24 $$J = \alpha^{T} E\alpha + \lambda \left( {R^{T} \alpha - 1} \right)$$

25 $$\frac{\partial J}{{\partial \alpha }} = 2E\alpha + \lambda R = 0$$

26 $$\alpha = \frac{{E^{ - 1} R}}{{R^{T} E^{ - 1} R}}$$

#### Accuracy of the model

In terms of forecast accuracy metrics, this paper refers to the traditional common model evaluation metrics: root mean square error (RMSE) and mean absolute percentage error (MAPE), and describes the forecast error evaluation metrics: CMAPE and CRMSE for compositional data^49^. The specific formulas are as follows.

27 $$CRMSE = \frac{1}{T - M}\sum\limits_{t = M + 1}^{T} {d_{s} \left( {x^{\left( t \right)} ,\hat{x}^{\left( t \right)} } \right)}$$

28 $$CMAPE = \frac{1}{T - M}\sum\limits_{t = M + 1}^{T} {\frac{{d_{s} \left( {x^{\left( t \right)} ,\hat{x}^{\left( t \right)} } \right)}}{{x_{s}^{\left( t \right)} }}}$$

### Framework for the study

**Figure 3:**
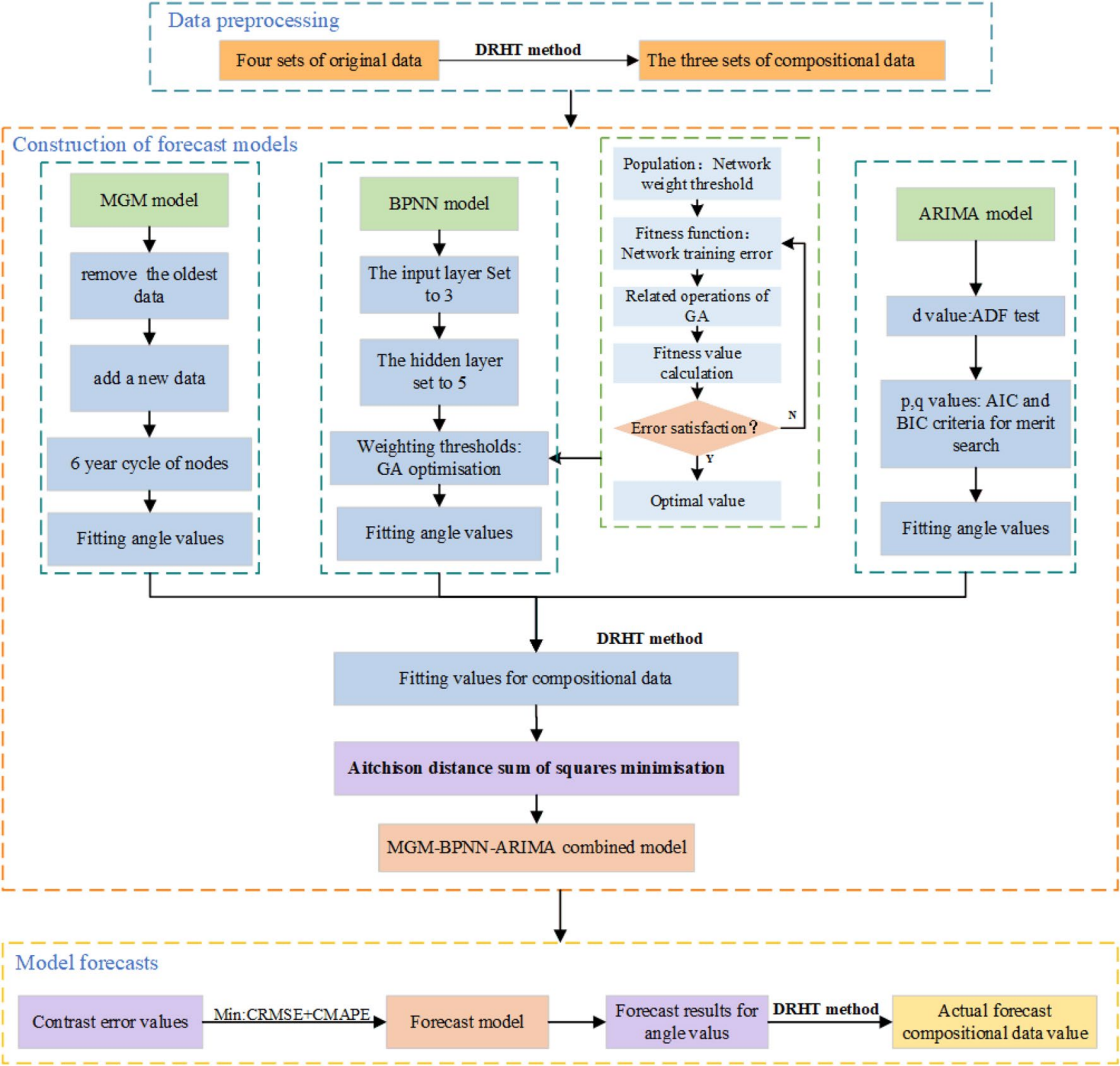
The methodological framework for this paper.

Considering the vectorial nature of the compositional data, to further improve the accuracy of the energy consumption structure forecast, this paper proposes a compositional binding model based on the theory of minimizing the sum of the squared Aitchison distance errors from the compositional data. In contrast, the study process in this paper is divided into three main steps: (1) Data pre-processing. The primary energy consumption structure is transformed into angle values (intermediate variables), that apply DRHT methods for compositional data. (2) Construction of forecast models. The MGM, BPNN, and ARIMA models were established respectively, the angle values under each model were fitted, and the weight values for each model were calculated, in which Atchison distance squared and minimization theory was applied, with inversion performed by the DRHT method, to obtain the forecast values for the compositional data. (3) Model forecasting. The optimized forecast model is elected by minimum CRMSE and CMAPE values, together with the DRHT method to back-transform the forecast obtained angle values to the actual forecast values. The concrete prediction framework is shown in Fig. 3.

## Model establishment and analysis

### Data

The research object of this paper is the energy consumption structure of China for 2000–2022, and the data are obtained from the China National Statistical Yearbook and National Bureau of Statistics. Moreover, the energy consumption structure covered in this paper is divided into four categories: coal, oil, natural gas, and other energy sources (like hydropower and wind energy). The specific structure of Chinese energy consumption during 2000–2022 is depicted in Table 1, which reveals that coal has long dominated the energy consumption structure, although there has been a more pronounced decreasing trend in the percentage of coal in recent years. The percentage of oil has also been falling each year. The percentage of clean energy (like natural gas) has increased significantly, and despite the continuous improvement and adjustment of the energy consumption structure in China, it is still unbalanced in general.

**Table 1:** The structure of energy consumption in China during 2000–2022.

Year	Coal	Oil	Natural Gas	Others	Year	Coal	Oil	Natural Gas	Others
2000	0.685	0.220	0.022	0.073	2012	0.685	0.170	0.048	0.097
2001	0.680	0.212	0.024	0.084	2013	0.674	0.171	0.053	0.102
2002	0.685	0.210	0.023	0.082	2014	0.658	0.173	0.056	0.113
2003	0.702	0.201	0.023	0.074	2015	0.638	0.184	0.058	0.120
2004	0.702	0.199	0.023	0.076	2016	0.622	0.187	0.061	0.130
2005	0.724	0.178	0.024	0.074	2017	0.606	0.189	0.069	0.136
2006	0.724	0.175	0.027	0.074	2018	0.590	0.189	0.076	0.145
2007	0.725	0.170	0.030	0.075	2019	0.577	0.190	0.080	0.153
2008	0.715	0.167	0.034	0.084	2020	0.569	0.188	0.084	0.159
2009	0.716	0.164	0.035	0.085	2021	0.560	0.185	0.089	0.166
2010	0.692	0.174	0.040	0.094	2022	0.562	0.179	0.084	0.175
2011	0.702	0.168	0.046	0.084	–	–	–	–	–

*Data from China National Statistical Yearbook and National Bureau of Statistics.

### Transformation of compositional data

Taking into account the peculiar circumstance that the energy consumption structure may have zero subcomponents, in this study, the DRHT approach can be applied to analyze the Chinese energy consumption structure during 2000–2022. Meanwhile, the MGM, BPNN, and ARIMA model are adopted as the benchmark model for the combined model. Before proceeding with the model, the data of the original consumption structure of Table 1 is first subjected to the DRHT method. In this paper, $\left( {y_{1} ,y_{2} ,y_{3} ,y_{4} } \right)$ are represented the four major components of the energy consumption structure, and $\left( {\theta_{1} ,\theta_{2} ,\theta_{3} } \right)$ are denoted as the angle values of the compositional data transformed with DRHT, and the specific angle values after conversion are presented in Table 2.

**Table 2:** Results of DRHT transformation of energy consumption structure from 2000 to 2022.

Year	$\theta_{2}$	$\theta_{3}$	$\theta_{4}$	Year	$\theta_{2}$	$\theta_{3}$	$\theta_{4}$
2001	1.062	1.408	1.277	2012	1.109	1.338	1.254
2002	1.065	1.412	1.280	2013	1.104	1.325	1.246
2003	1.079	1.413	1.295	2014	1.097	1.317	1.228
2004	1.082	1.412	1.291	2015	1.078	1.311	1.217
2005	1.110	1.409	1.295	2016	1.069	1.303	1.202
2006	1.114	1.399	1.295	2017	1.061	1.284	1.193
2007	1.120	1.390	1.293	2018	1.056	1.268	1.180
2008	1.121	1.377	1.277	2019	1.050	1.258	1.169
2009	1.124	1.374	1.275	2020	1.049	1.249	1.161
2010	1.106	1.359	1.259	2021	1.049	1.238	1.151
2011	1.116	1.345	1.277	2022	1.057	1.246	1.139

### Construction of the single model

Based on the data in Table 2, the MGM (1,1) model is utilized to forecast the three groups of angle values $\left( {\theta_{2} ,\theta_{3} ,\theta_{4} } \right)$, in which the loop node of the MGM model used in this paper is set to 6 through multiple fitting, meaning that the data from the previous six years can forecast the angle value for the upcoming year. Since the MGM(1,1) model is applied to forecast for all three sets of data $\left( {\theta_{2} ,\theta_{3} ,\theta_{4} } \right)$, and the years forecasted span for 2000–2022, the forecasts for 51 angle values can be obtained. Furthermore, the forecasted angle values are inverted to the compositional data, to derive the fitted values for each component of energy consumption structure from 2006 to 2022, as summarized in Table 3.

**Table 3:** The fitting results of the single model for 2006–2022.

Year	MGM				BPNN				ARIMA			
	y_1_	y_1_	y_3_	y_4_	y_1_	y_2_	y_3_	y_4_	y_1_	y_2_	y_3_	y_4_
2006	0.730	0.176	0.023	0.070	0.721	0.174	0.027	0.078	0.717	0.180	0.026	0.078
2007	0.737	0.165	0.027	0.071	0.728	0.164	0.031	0.078	0.725	0.168	0.030	0.078
2008	0.735	0.159	0.031	0.075	0.714	0.173	0.034	0.079	0.719	0.170	0.033	0.079
2009	0.725	0.157	0.036	0.082	0.708	0.171	0.038	0.083	0.704	0.171	0.037	0.088
2010	0.718	0.155	0.039	0.088	0.704	0.172	0.039	0.085	0.708	0.166	0.037	0.089
2011	0.702	0.158	0.043	0.098	0.681	0.174	0.044	0.101	0.688	0.171	0.044	0.098
2012	0.689	0.169	0.049	0.093	0.675	0.179	0.050	0.095	0.687	0.175	0.050	0.088
2013	0.680	0.171	0.053	0.096	0.670	0.165	0.052	0.113	0.679	0.169	0.051	0.101
2014	0.666	0.172	0.058	0.104	0.652	0.173	0.057	0.118	0.667	0.170	0.057	0.106
2015	0.653	0.171	0.061	0.115	0.640	0.171	0.060	0.128	0.648	0.175	0.059	0.118
2016	0.625	0.183	0.062	0.130	0.627	0.181	0.063	0.129	0.629	0.186	0.061	0.125
2017	0.607	0.190	0.065	0.138	0.607	0.188	0.066	0.140	0.611	0.190	0.064	0.135
2018	0.588	0.195	0.070	0.146	0.595	0.185	0.073	0.147	0.598	0.187	0.074	0.141
2019	0.573	0.195	0.079	0.153	0.575	0.183	0.076	0.166	0.584	0.185	0.081	0.150
2020	0.561	0.191	0.087	0.162	0.565	0.182	0.079	0.174	0.570	0.188	0.084	0.158
2021	0.552	0.189	0.091	0.167	0.556	0.180	0.082	0.182	0.561	0.187	0.088	0.164
2022	0.546	0.185	0.094	0.174	0.552	0.176	0.084	0.188	0.554	0.181	0.093	0.171

*Due to the different nodes chosen for the models, only the results of the fit for the three models for the common years are shown.

Regarding the time series prediction of energy consumption structures using the BPNN model, in this paper, the specific energy consumption substructures of three consecutive years are used as inputs to the neural network to predict the substructures of the following year through multiple fitting. Since there are three sets of angular values after DRHT conversion, three different network models need to be constructed. Regarding the setting of the initial parameters of the neural network, its training function is set to train with a maximum number of iterations of 1000 and an error threshold of 1e-6. For the determination of the hidden layers of neural networks, the hidden layers of the three networks are chosen to be set to five layers in this paper by comparing the models trained with multiple layers. To further improve the prediction accuracy and generalization ability of the BPNN model, the genetic algorithm (GA) is employed in this paper to optimize the weights and thresholds of the BPNN model. For the initial parameters of GA, the selection probability is set to 0.09, the crossover probability is set to 0.4, and the variation pattern is nonUnif Mutation. Then, the trained GA-BPNN model is employed to forecast three sets of angle values. These are loosely related to the values in Table 3 previously. Finally, the predicted angular values are inverse transformed to the compositional data to obtain the fitted values for the components of the energy consumption structure, which are summarized in Table 3.

According to the data in Table 2, three independent ARIMA models need to be constructed in this paper to predict the three data sets separately. based on the information criterion of minimization of AIC and BIC to find the optimal parameter values of the three ARIMA models, and the three ARIMA models finally identified for adoption as ARIMA(1,0,2), ARIMA(1,1,0) and ARIMA(0,1,0). Meanwhile, the fitting goodness of fit of all three models is above 0.85, which is a good fit. The fitting results of the above three ARIMA models values after inversion with the compositional data are demonstrated in Table 3.

### Optimal model selection

**Figure 4:**
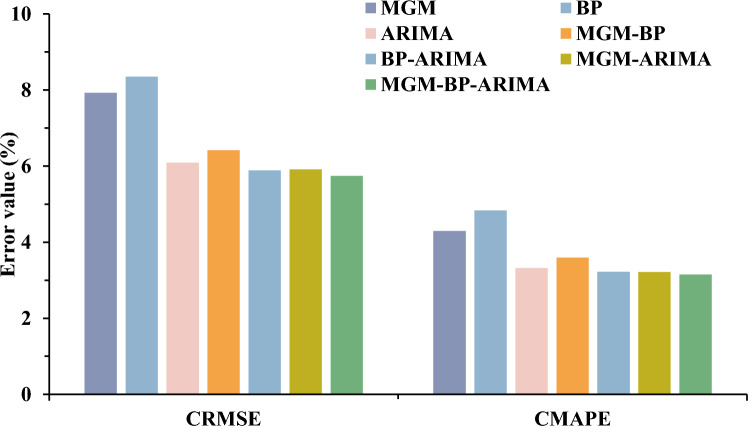
Comparison of CRMSE and CMAPE values for each model.

In this paper, the three monomial models constructed above are used as benchmark models for compositional prediction, simultaneously the weights of the combined MGM-BPNN-ARIMA model are derived from the theory of minimization of squared Atchison distances on compositional data. To additionally select the optimal joint model, the corresponding joint model is also constructed in this paper based on any two of the single models mentioned above. The weights and error values for the specific joint model are given in Table 4. Next, the CMAPE and CRMSE values of each combined model are compared and the model with the lowest error is chosen as the forecast model. Considering the inconsistent data nodes used by each model, the values from 2006 to 2022 are presented as the basic values for the weight assignment and model error comparison in this paper. Moreover, Fig. 4 compares the CRMSE and CMAPE values for all potential merger models.

**Table 4:** Combined weight allocation and error value summary results of each model.

Model	Weight	CRMSE (%)	CMAPE (%)
MGM(1,1)	–	7.928	4.296
BPNN	–	8.352	4.833
ARIMA	–	6.092	3.323
MGM-BPNN	(0.514, 0.487)	6.416	3.593
BPNN-ARIMA	(0.268, 0.732)	5.885	3.226
MGM-ARIMA	(0.165, 0.835)	5.914	3.214
MGM-BPNN-ARIMA	(0.181, 0.275, 0.544)	5.739	3.150

Table 4 sums up the error values (CRMSE and CMAPE) of each model and the results of the weight assignment of the combined model. It can be stated that the error values of the combined models are all less than the single model, in which the ARIMA model is the single model with the lowest error, and the combination of the benchmark models performs well, with CRMSE values below 6% and CMAPE values below 3.25%. And the best prediction is achieved by the MGM-BPNN-ARIMA combination model. The weight value of this combined model is (0.181,0.275,0.544), which predicted the CRMSE value of 5.739% and the CMAPE value of 3.150%. Compared to the ARIMA model, which has the smallest error value among the individual models, its CMAPE value is reduced by 0.173%, and its CRMSE value is reduced by about 0.353%, and compared to the combined BPNN-ARIMA and MGM-ARIMA model based on the ARIMA model, the CMAPE values are reduced by 0.076% and 0.064%, and the CRMSE values are reduced by about 0.146% and 0.175%. It implies that the combined MGM-BPNN-ARIMA model constructed improves the forecast accuracy. Moreover, it also further illustrates that the forecast of the compositional data based on the Atchison distance squared and minimization theory has obvious advantages, as it completely utilizes the internal structural features of the compositional data for the study.

## Forecast results and discussion

**Figure 5:**
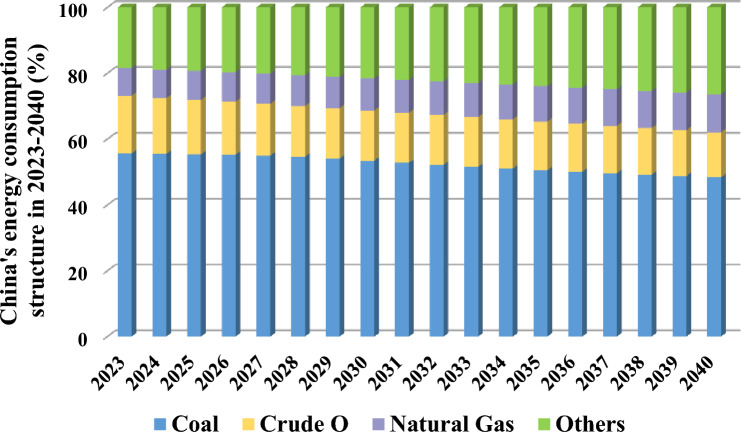
The energy consumption structure in China for 2023–2040.

Based on the values of China’s energy consumption structure during 2000–2022, the model (the DRHT transformed MGM-BPNN-ARIMA combination model) with the lowest CRMAE and CMAPE values is adopted in this paper, to forecast the energy consumption structure of China for 2023–2040. The forecast results of the specific sub-structure percentages for the four categories of energy consumption structure are shown in Table 5, while the trends of each type of energy consumption are depicted in Fig. 5.

**Table 5:** Forecast results of the structure of energy consumption in China for 2023–2040.

Year	Coal	Oil	Natural gas	Others	Year	Coal	Oil	Natural gas	Others
2023	0.557	0.175	0.085	0.184	2032	0.522	0.152	0.102	0.225
2024	0.555	0.169	0.086	0.189	2033	0.516	0.151	0.104	0.229
2025	0.553	0.166	0.088	0.193	2034	0.510	0.150	0.106	0.234
2026	0.553	0.161	0.090	0.196	2035	0.505	0.148	0.108	0.239
2027	0.550	0.157	0.092	0.201	2036	0.500	0.146	0.109	0.244
2028	0.546	0.154	0.094	0.206	2037	0.496	0.144	0.111	0.249
2029	0.541	0.153	0.096	0.211	2038	0.492	0.142	0.113	0.254
2030	0.535	0.152	0.098	0.215	2039	0.488	0.139	0.115	0.259
2031	0.528	0.152	0.100	0.220	2040	0.484	0.136	0.116	0.263

**Figure 6:**
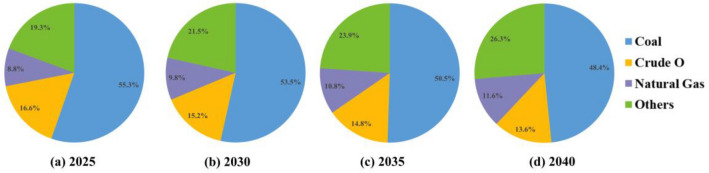
Comparison of China’s energy consumption structure in 2025, 2030, 2035 and 2040.

As indicated in Table 5 and Fig. 5, the future energy consumption structure of China will be adjusted and improved, in which the proportion of coal consumption will keep decreasing, and remain at 53.5% in 2030 and 48.4% in 2040, which means that coal consumption will still hold a major position in China’s energy consumption structure. Simultaneously, the share of oil consumption will also decrease, from about 17.5% in 2023 to 13.6% in 2040, so the proportion of fossil energy consumption will show an obvious declining trend, further indicating the optimized adjustment of China’s energy consumption structure in the future. Meanwhile, the proportion of natural gas consumption will maintain an upward trend, rising substantially from 8.5% in 2023 to 11.6% in 2040. And the proportion of other clean energy (e.g., wind power and hydropower) will reach 21.5% in 2030, 23.9% in 2035, and 26.3% in 2040, a significant increase from 18.4% in 2023. To further compare the forecast results with the actual policy goals, a concrete comparison of the energy consumption structure of China in 2025, 2030, 2035 and 2040 is shown in Fig. 6. In accordance with the above statistics, there is a rapid development of non-fossil energy, and its share in the energy consumption structure is increasing each year, but the entire energy consumption structure is still in a state of imbalance, which also means that China’s energy consumption structure still needs to be adjusted and optimized more.

1. *Coal*. Coal will remain a substantial part of China’s energy consumption structure in 2023–2040, but its share shows a decreasing trend, falling from 55.7% in 2023 to 48.4% in 2040. However, as a major energy consumer, China’s total energy consumption has always been large and coal is still used to some extent at peak consumption levels. Therefore, China should stick to the objective of exploring new energy sources to alternative coal consumption, so that coal gradually loses its dominance in energy consumption.
2. *Oil*. From 2023 to 2040, China’s oil share shows a clear downward trend, from 17.5% in 2023 to 13.6% in 2040. Therefore, there is a downward trend for fossil energy (i.e., oil and coal), but the energy consumption structure in China will be dominated by them over time. It is essential for the Chinese government to take measures to develop non-fossil energy sources and reduce oil consumption, thus promoting an optimal transformation of the energy consumption structure.
3. *Natural gas*. The “Strategy for the Energy Production and Consumption Revolution (2016–2030)” mentions that by 2030, China will reach a natural gas consumption share of about 15%. However, gas consumption is only 9.8% in 2030 and 11.6% in 2040, falling short of the proposed policy target. As such, it is critical to make effective policy adjustments to increase the production and supply of natural gas and thus promote its substitution for conventional elevated-carbon fossil energy sources.
4. *Others*. The “Action Plan to Achieve Carbon Peak by 2030” issued that during “the 14th Five-Year Plan (2020–2025)”, By 2025, China will have made major strides in the optimization and adjustment of its energy structure, and the percentage of non-fossil energy consumption will be close to 20%. During “the Tenth Five-Year Plan (2026–2030)”, the percentage of non-fossil energy consumption will be more increased, and by 2030, the percentage of non-fossil energy consumption will reach about 25%. However, the forecasted conclusions indicate that the percentage of non-fossil energy consumption in 2030 would only be 21.5%, falling short of the 2030 policy aim, and only corresponding with the Chinese the administration’s 2025 policy target. As a result, China still needs to step up its energy reform efforts, accelerate the development of renewable energy technologies such as wind and solar power, and grow the clean energy industry.

To further support the improvement and modification of China’s energy consumption structure, the following recommendations are made. First, a more detailed and clear development roadmap should be drawn up to ensure that the policy goals set can be promoted in a reasonable and orderly manner. Second, high-tech development should be vigorously developed to speed up the transformation of the industrial structure. Second, we should develop high-tech technologies and accelerate the transformation of industrial structures. For example, improving the energy utilization efficiency of key industries with “high energy consumption” and “elevated emissions” (e.g., the iron and steel industry), to achieve the ultimate “coal reduction”. Third, the development of a diverse energy landscape should be actively encouraged, which implies expanding the growth of clean energy sources, such as water and wind power, and the progressive and orderly replacement of fossil energy consumption by clean renewable energy consumption, as with coal. Finally, public awareness of green and low-carbon development should be increased, and green consumption by all should be encouraged.

## Conclusions

The energy consumption structure is fundamentally a holistic system with a disjoint internal structure, which implies that its constituent parts are non-negative and add up to one. However, due to the lack of sufficient excavation of information on the energy consumption structure, few scholars have conducted research in this area. At the same time, classic time series forecasting methods determine the percentages of each component independently, ignoring structural integrity and failing to thoroughly examine internal development trends. As a result, this paper incorporates compositional data into the energy consumption structure and evaluates the energy consumption structure as a whole system. This not only meets the numerical restrictions of the components (non-negative and constant), but also effectively displays the intrinsic development trend of each component behind the system. In this paper, we use historical data to forecast the trend of China’s energy consumption structure from 2000 to 2022. In terms of forecasting model selection, this paper proposes a joint MGM-BPNN-ARIMA forecasting model with the best predictive performance based on the traditional single model to forecast the evolution of China’s energy consumption structure during 2023–2040.

With the overall objectives of “carbon peaking” and “carbon neutrality”, the Chinese authorities have taken a series of practical steps to optimize the energy structure, and have also set policy goals for the energy consumption structure of China. The predictive studies presented in this paper can, to some extent, test whether the policy objectives for China’s energy consumption structure can be achieved as expected. Based on the combined MGM-BPNN-ARIMA model predictions after the DRHT conversion constructed in this paper, it is evident that the Chinese energy consumption structure is still in a non-reciprocal state during 2023–2040, with the coal already dominating the energy consumption structure but gradually declining in importance. The percentage of non-fossil energy consumption will be 19.3% in 2025, 9.8% for natural gas and 21.5% for non-fossil energy in 2030, which is considerably different from the policy target, but the share of clean energy consumption has increased to 31.3%.

This paper incorporates compositional data into the study of China’s energy consumption structure forecast, which fully considers the overall structure and the internal characteristics required. However, this predictive approach is mainly based on historical data and does not take into account various effects such as actual polarization. Therefore, in the sequel, it is necessary to integrate the essential affecting forces of the energy consumption structure with the theory of compositional data to construct a multi-factorial dynamic predictive model.

## Data Availability

The datasets used and analyzed during the current study are available from the corresponding author on reasonable request.

## References

[1] Tagne RFT, Dong X, Anagho SG, Kaiser S, Ulgiati S (2021). Technologies, challenges and perspectives of biogas production within an agricultural context. The case of China and Africa. Environ. Dev. Sustain..

[2] Shi M (2022). Forecast of China's carbon emissions under the background of carbon neutrality. Environ. Sci. Pollut. Res. Int..

[3] Singh MK, Mukherjee D (2018). Drivers of greenhouse gas emissions in the United States: Revisiting STIRPAT model. Environ. Dev. Sustain..

[4] Xie P, Xu Y, Tan X, Tan Q (2023). How does environmental policy stringency influence green innovation for environmental managements?. J. Environ. Manag..

[5] Ge Y, Yuan R, Liao H (2023). Decoupling analysis and peak projection of manufacturing CO(2) emissions from the perspective of investment. Environ. Dev. Sustain..

[6] Yu Z (2020). Dynamic changes, spatiotemporal differences and factors influencing the urban eco-efficiency in the lower reaches of the Yellow River. Int. J. Environ. Res. Public Health.

[7] Wang T, Liu J, Xu Y (2022). Primary energy consumption structure and the influencing factors in China: An income decomposition and post-economic crisis era perspective. Environ. Sci. Pollut. Res. Int..

[8] Bilgen S (2014). Structure and environmental impact of global energy consumption. Renew. Sustain. Energy Rev..

[9] Tang L, Wang S, He K, Wang S (2015). A novel mode-characteristic-based decomposition ensemble model for nuclear energy consumption forecasting. Ann. Oper. Res..

[10] Wang X, Luo D, Zhao X, Sun Z (2018). Estimates of energy consumption in China using a self-adaptive multi-verse optimizer-based support vector machine with rolling cross-validation. Energy.

[11] Barak S, Sadegh SS (2016). Forecasting energy consumption using ensemble ARIMA–ANFIS hybrid algorithm. Int. J. Electr. Power Energy Syst..

[12] Bin Shams M, Haji S, Salman A, Abdali H, Alsaffar A (2016). Time series analysis of Bahrain's first hybrid renewable energy system. Energy.

[13] Jiang S, Yang C, Guo J, Ding Z (2018). ARIMA forecasting of China’s coal consumption, price and investment by 2030. Energy Sources Part B Econ. Plan. Policy.

[14] Akram J, Dina J, Amid M, Mohammadreza K (2019). An auto regressive integrated moving average (ARIMA) model for prediction of energy consumption by household sector in Euro area. AIMS Energy.

[15] Ding S, Li R, Wu S, Zhou W (2021). Application of a novel structure-adaptative grey model with adjustable time power item for nuclear energy consumption forecasting. Appl. Energy.

[16] Yuan C, Liu S, Fang Z (2016). Comparison of China's primary energy consumption forecasting by using ARIMA (the autoregressive integrated moving average) model and GM(1,1) model. Energy.

[17] Li S, Yang X, Li R (2019). Forecasting coal consumption in India by 2030: Using linear modified linear (MGM-ARIMA) and linear modified nonlinear (BP-ARIMA) combined models. Sustainability.

[18] Ma M, Wang Z (2019). Prediction of the energy consumption variation trend in South Africa based on ARIMA, NGM and NGM-ARIMA models. Energies.

[19] He Y, Zheng Y, Xu Q (2019). Forecasting energy consumption in Anhui province of China through two Box-Cox transformation quantile regression probability density methods. Measurement.

[20] Wang WW, Liu X, Zhang M, Song XF (2014). Using a new generalized LMDI (logarithmic mean Divisia index) method to analyze China's energy consumption. Energy.

[21] Peduzzi PN, Hardy RJ, Holford TR (1980). A stepwise variable selection procedure for nonlinear regression models. Biometrics.

[22] Yu S-W, Zhu K-J (2012). A hybrid procedure for energy demand forecasting in China. Energy.

[23] Chauhan VK, Dahiya K, Sharma A (2019). Problem formulations and solvers in linear SVM: A review. Artif. Intell. Rev..

[24] Kim TY, Cho SB (2019). Predicting residential energy consumption using CNN-LSTM neural networks. Energy.

[25] Xia C, Wang Z (2020). Drivers analysis and empirical mode decomposition based forecasting of energy consumption structure. J. Clean. Prod..

[26] Chai J, Liang T, Lai KK, Zhang ZG, Wang S (2018). The future natural gas consumption in China: Based on the LMDI-STIRPAT-PLSR framework and scenario analysis. Energy Policy.

[27] Liu Y, Jiang Y, Liu H, Li B, Yuan J (2021). Driving factors of carbon emissions in China’s municipalities: A LMDI approach. Environ. Sci. Pollut. Res..

[28] He Y, Chen Y, Zhang W, Wang Y (2022). Optimizing energy consumption structure in Chongqing of China to achieve low-carbon and sustainable development based on compositional data. Sustain. Energy Technol. Assess..

[29] Wei Y, Wang Z, Wang H, Li Y (2021). Compositional data techniques for forecasting dynamic change in China’s energy consumption structure by 2020 and 2030. J. Clean. Prod..

[30] Qian W, Zhang H, Sui A, Wang Y (2022). A novel adaptive discrete grey prediction model for forecasting development in energy consumption structure—From the perspective of compositional data. Grey Syst. Theory Appl..

[31] Aitchison J (1982). The statistical analysis of compositional data. J. Roy. Stat. Soc. Ser. B (Methodological).

[32] Egozcue JJ, Pawlowsky-Glahn V, Mateu-Figueras G, Barceló-Vidal C (2003). Isometric logratio transformations for compositional data analysis. Math. Geol..

[33] Hwang D (2005). A data integration methodology for systems biology: Experimental verification. Proc. Natl. Acad. Sci..

[34] Zhao, L., Ping, Y. & Luo, Y. In *2018 2nd International Conference on Applied Mathematics, Modelling and Statistics Application (AMMSA 2018).* 215–219 (Atlantis Press).

[35] Wei YG, Wang ZC, Wang HW, Li Y, Jiang ZY (2019). Predicting population age structures of China, India, and Vietnam by 2030 based on compositional data. PLoS One.

[36] Nishio I (2022). Compositional data analysis (CoDA) of clinopyroxene from abyssal peridotites. Geochem. Geophys. Geosyst..

[37] Zhang K, Yin K, Yang W (2022). Predicting bioenergy power generation structure using a newly developed grey compositional data model: A case study in China. Renew. Energy.

[38] Wang, D., Gan, J., Mao, J., Chen, F. & Yu, L. Forecasting power demand in China with a CNN-LSTM model including multimodal information. *Energy* **263**. 10.1016/j.energy.2022.126012 (2023).

[39] Liu S, Zeng B (2022). Combined prediction of clean energy consumption in China based on the nonlinear programming model. Math. Problems Eng..

[40] Ratchagit M, Xu H (2022). A two-delay combination model for stock price prediction. Mathematics.

[41] Meng M, Niu DX, Shang W (2014). A small-sample hybrid model for forecasting energy-related CO2 emissions. Energy.

[42] Shang ZH (2022). A novel model based on multiple input factors and variance reciprocal: Application on wind speed forecasting. Soft Comput..

[43] Filzmoser P, Hron K (2009). Correlation analysis for compositional data. Math. Geosci..

[44] Greenacre M (2021). Compositional data analysis. Ann. Rev. Stat. Appl..

[45] Wang H, Liu Q, Mok HMK, Fu L, Tse WM (2007). A hyperspherical transformation forecasting model for compositional data. Eur. J. Oper. Res..

[46] Deng J (2002). The Fundamental of Grey Theory.

[47] Li, J., Cheng, J.-H., Shi, J.-Y. & Huang, F. In *Advances in Computer Science and Information Engineering.* (eds David Jin & Sally Lin) 553–558 (Springer).

[48] Li SY, Li RR (2017). Comparison of forecasting energy consumption in Shandong, China using the ARIMA model, GM model, and ARIMA-GM model. Sustainability.

[49] Wei Y, Wang Z, Wang H, Yao T, Li Y (2018). Promoting inclusive water governance and forecasting the structure of water consumption based on compositional data: A case study of Beijing. Sci. Total Environ..

